# Validation and development of a shorter version of the resilience scale RS-11: results from the population-based KORA–age study

**DOI:** 10.1186/2050-7283-1-25

**Published:** 2013-11-22

**Authors:** Alexander von Eisenhart Rothe, Markus Zenger, Maria Elena Lacruz, Rebecca Emeny, Jens Baumert, Sibylle Haefner, Karl-Heinz Ladwig

**Affiliations:** Helmholtz Zentrum München, German Research Center for Environmental Health, Institute of Epidemiology II, Ingolstädter Landstr. 1, Neuherberg, 85764 Germany; Department of Medical Psychology and Medical Sociology, University of Leipzig, Philipp-Rosenthal-Str. 55, Leipzig, 04103 Germany; Institute of Clinical Epidemiology, Martin-Luther University Halle-Wittenberg, Halle (Saale), Germany

**Keywords:** Resilience, Psychometrics, Mental health, Aging

## Abstract

**Background:**

The aim of this study was to assess reliability and validity of the Resilience Scale 11 (RS-11) and develop a shorter scale in a population-based study.

**Methods:**

The RS-11 scale was administered to 3942 participants (aged 64 – 94 years) of the KORA-Age study. To test reliability, factor analyses were carried out and internal consistency (Cronbach’s α) was measured. Construct validity was measured by correlating scores with psychological constructs. The criterion for a shorter scale was a minimum internal consistency of .80. Shorter models were compared using confirmatory factor analysis. Sensitivity and specificity of RS-5 to RS-11 was analyzed.

**Results:**

Factor analysis of the RS-11 gave a 1-factor solution. Internal consistency was α = .86. A shorter version of the scale was developed with 5 items, which also gave a 1-factor solution and showed good validity. Internal consistency of this shorter scale: Resilience Scale 5 (RS-5) was α = .80. Sensitivity and specificity of RS-5 compared with RS-11 were .79 and .91 respectively. Both scales correlated significantly in expected directions with related constructs.

**Conclusions:**

The RS-11 and the RS-5 are reliable, consistent and valid instruments to measure the ability of elderly individuals to successfully cope with change and misfortune.

**Electronic supplementary material:**

The online version of this article (doi:10.1186/2050-7283-1-25) contains supplementary material, which is available to authorized users.

## Background

“Resilience” (or psychosocial stress-resistance) is the term used to describe a person’s capability to adapt positively to adverse conditions (Luthar et al. 2000).

Studies suggest that resilient elderly have better mental health (Hardy et al. 2002; Wagnild 2003), less depression in late life (Mehta et al. 2008) and that resilience correlates with self-rated successful aging (Lamond et al. 2008), better health outcomes (Smith 2006) and survival of the elderly (Shen & Zeng 2010). Furthermore, it has been demonstrated a tendency that resilient persons suffering from diabetes have better glycosylated haemoglobin (Steinhardt et al. 2009; Yi et al. 2008).

The “Resilience Scale”, (Wagnild & Young 1993), describes resilience as being a positive personality trait which facilitates personal adaptation, i.e. coping with change or misfortune. Construct validity for versions of the Resilience scale has been measured by correlations with depression, physical health, life satisfaction, morale, anxiety, stress and health promoting activities (Heilemann et al. 2003; Ahern et al. 2006; Abiola & Udofia 2011).

In this study we aimed to assess the reliability and validity of the German version of the resilience scale (RS-11) (Schumacher et al. 2005) and subsequently we aimed to develop a shorter version of the scale in a large, elderly German population. In large cohort studies it is not possible to include many lengthy questionnaires, this holds particularly true in studies of older individuals. Thus, there is an urgent need for short-scales, particularly with regard to resilience, for which population-based research is lacking. The inclusion of the concept in large, population-based studies will allow for analysis of the effects of fostering resilience, and analysis into how this concept might fit within the ever increasingly understood mechanisms of psychosocial factors on health. The development of an abbreviated version should encourage the inclusion of the instrument in large cohort studies and reduce missing data. The main hypothesis to be tested is that the short version of the RS-11, which must have a good internal consistency, will demonstrate acceptable model fit indices as computed with confirmatory factor analysis (CFA).

## Methods

### Sample

Data was taken from the KORA (Cooperative Research in the Region of Augsburg) - Age study (2008–2009) (Lacruz et al. 2010; Peters et al. 2011), which is a follow-up study of older participants from the population-based MONICA(MOnitoring Trends and Determinants in CArdiovascular Disease )/KORA Augsburg studies. The local authorities approved the study and all participants provided written informed consent. The study protocol was submitted and approved by the Ethical Committee of the Bavarian medical association (Ethik- Kommission Nr. 08064). More information about the study design of KORA is given elsewhere (Holle et al. 2005).

The KORA-Age study involved a follow-up health questionnaire administered to all participants of the cohort who were born between 1915 and 1943 (n = 4565; response rate = 76%); a telephone interview to determine multimorbidity and mental health status of the participants (N = 4127; response rate = 69%), and medical examinations and personal interviews to a sub-sample of the cohort (n = 1079; response rate = 54%).

The RS-11 was administered in the telephone interview. We excluded participants who did not answer the telephone interview themselves (n = 185) and those with cognitive impairment (n = 146) as determined with TICS-m (Telephone interview for cognitive status – modified) (Breitner & Welsh 1995; Crooks et al. 2005; Perneczky 2003) using a cut off value of 27 (Knopman et al. 2010).

Eighty-four participants with missing values on the resilience scale were excluded. Excluded participants were more likely to be older (OR = 4.54, 95% CI: 2.55 – 8.09), reporting any disability (OR = 3.15, 95% CI: 1.96 – 5.08), not living alone (OR = 2.17, 95% CI: 1.41 – 3.35), to have low self-rated health (OR = 2.25, 95% CI: 1.46 – 3.49), low well-being (OR = 2.79, 95% CI: 1.1 – 7.08) and depressed mood (OR = 3.64, 95% CI: 1.43 – 9.3). No significant differences were found in distributions of sex, loneliness or anxiety.

Thus, the analyzed study sample consisted of 3 712 participants (52% women, 48% men). Age ranged from 64 to 94 years (median = 72; mean = 73; SD ± 5.8).

### Measures

Resilience was measured using the German RS-11 scale (see Additional file 1) (Schumacher et al. 2005). The RS-25 has an internal consistency of .91. The scale has a 2-factor structure. These are titled “Personal Competence” and “Acceptance of Self and Life”. Construct validity was assessed by correlations with depression (r = −.37), physical health (r = −.26), life satisfaction (r = .30) and morale (r = .28) (Wagnild & Young 1993). Since their development, RS scales have been validated in a wide range of populations including adolescents (Hunter & Chandler 1999; Black & Ford-Gilboe 2004) and older populations (Wagnild 2003) and in many different ethnicities and languages, reporting internal consistencies ranging from .72 to .94 (Ahern et al. 2006; Abiola & Udofia 2011; Ruiz-Parraga et al. 2012; Damasio et al. 2011).

The abbreviated German RS-11 was developed in 2,031 participants of a large community sample of the German population (aged 14 to 95). A 1-factor structure was reported. The internal consistency of this scale was shown to be excellent with α = .91 (Schumacher et al. 2005). The RS-11 score is calculated by summing up the values 1 (=strongly disagree) to 7 (=strongly agree) across all eleven questions. The resulting sum score thus ranges from 11 to 77, where higher scores represent greater resilience. In the present analysis, subjects in the top third of the resilience scale distribution were defined as resilient.

Depression was measured using the GDS-15 (Geriatric Depression Scale) from Sheikh and Yesavage (Sheikh & Yesavage 1985). This scale is comprised of 15 dichotomous items. In this study, participants with a score equal to or above 10 were classified as depressed.

The German version of the Generalized Anxiety Disorder scale (GAD-7) was used to determine feelings of anxiousness. The scale consists of 7 items on a 4-point Likert scale. The resulting score ranges from 0 to 21 (Spitzer et al. 2006). Participants with a score above or equal to 10 were classified as anxious, following (Kroenke et al. 2007).

Well-being was measured using the WHO-Five Well-being Index. This is a five-item questionnaire on a 6-point Likert scale (0 = not present to 5 = constantly present) which is translated into a score ranging from 0 to 100. Higher scores mean higher well-being. Participants with a score below 25 screened negative for well-being (Bech 2004).

Social network was assessed with the “Social Network Index” (SNI). The SNI is based on the “social network scale” (“Berkman-Index”) of the Alameda-County-Study (Berkman 1982) and is detailed and reproduced in the WHO: MONICA Psychosocial Optional Study (MOPSY) (WHO 1989). The SNI collects information on 12 types of social relationships. The scores of this index range from 1 to 4, with higher scores representing greater social network (WHO 1989). A dichotomous outcome was built to determine high vs. low social network.

Loneliness was measured by a shortened, German version of the UCLA-Loneliness-Scale which assesses subjective feelings of loneliness or social isolation (Russell et al. 1980). The scale consists of 12 items leading to a score ranging from 12 to 48. Higher scores indicate greater degrees of loneliness (Russell 1996). Subjects in the top third of the Loneliness-scale distribution were classified as being lonely.

Self-rated health was measured by a one-item question: respondents were asked, “How would you rate your current state of health?” Those who answered “very good” or “good” were classified as having good self-rated health.

Physical disability was measured using the Health Assessment Questionnaire Disability Index (HAQ-DI) (Bruce & Fries 2003). The outcome of the index is continuous and ranges from 0 to 3, where a score of 0 to1 represents mild/moderate disability, 1 to 2 moderate and 2 to 3 severe disability. Respondents with scores = 0 were classified as reporting no disability, and those with scores > 0 being classified as reporting any disability.

An age-specific median-split variable was determined (participants aged 72 years or older were classified as older). Age was additionally stratified in groups of five years. Due to small numbers in the oldest groups, these were summarized together. Thus, the four categories were defined as follows: 64–69, 70–74, 75–79 and 80 + .

### Statistical analyses

#### Descriptive analysis

The distribution of the RS-11 score was found to be non-normal (p = 0.01), using the Shapiro-Wilk test, even after taking the logarithm transformation. The Mann – Whitney U and Kruskal-Wallis tests were used to compare continuous variables with two, or more than two groups, respectively.

#### Validation of RS-11

The study population was split into two equal sized random samples. No significant differences were found between both random samples regarding all items of the questionnaire and socio-demographic variables (all p-values > .07).

In the first random sample (N = 1,856), an exploratory factor analysis (EFA) of the RS-11 was conducted using the principal axis factors method (extracting factors via the Kaiser criterion) and internal consistency (Cronbach’s α), average variance extracted (AVE) and composite reliability (CR) were calculated to explore the reliability and validity of RS-11 in an older population. To assess construct validity the scale was correlated (Spearman coefficient) with variables related to the construct of resilience, based on previous studies (Wagnild & Young 1993; Wagnild 2009). In the second random sample (N = 1,856), a CFA was computed to test the unidimensional structure reported in a population-based study (Schumacher et al. 2005).

#### Development and validation of a shorter scale

The main criteria for the shortened scale is a good Cronbach’s alpha value (≥.80) (Gliem & Gliem 2003). Although the originally postulated 2-factor structure is questionable (Schumacher et al. 2005; Windle et al. 2011), it is preferable that the new scale contains at least one item from each of the originally postulated dimensions. The “alphamax” macro was used (Hayes 2005) to establish combinations of items which result in a good Cronbach’s alpha (≥.80). In the second random sample, CFA was used to compare potential abbreviated questionnaire models. The CFA models were estimated with the maximum likelihood method approach, and the following fit indices were used: CMIN/DF, CFI, GFI, RMSEA, TLI and AIC.

Additional analyses were conducted to test the invariance of the model across gender and age using multi-group CFA. Measurement invariance was tested in three steps using first the configural model (no constraints), then a metric invariant model (with item loadings constraint to be equal across groups), and then a scalar invariant model (with item loadings and item intercepts simultaneously constraint to be equal across groups) (Gregorich 2006). Following the hierarchy of these nested models, they were compared to each other. Since the < chi > 2 statistic has often been criticized for its sensitiveness to the sample size, we focused mainly on ΔCFI and ΔRMSEA as indicators in the comparison of models. Values smaller than .01 indicate invariance of the models (Cheung & Rensvold 2002).

Furthermore, the chosen model was subjected to a sensitivity/specificity analysis in the full dataset, to calculate to what extent participants would be categorized as resilient when considering the top tertiles of both sum score distributions to be resilient. The Youden’s index was calculated for subgroups of related sociodemographic and psychological variables. A score of above 0.6 indicates a sufficient ability to discriminate (Hilden & Glasziou 1996). The shortened scale should have a satisfactory Youden’s Index when compared to the original scale, and should correlate strongly with the RS-11. Additionally, AVE and CR were measured.

For all analyses, p-values less than .05 were considered to be statistically significant. Statistical analyses were performed with the statistical software package SAS (Version 9.1, SAS-Institute Inc., Cary, NC, USA), and AMOS 18 (Arbuckle 2009).

## Results

### Descriptive analysis

In the whole study population (n = 3 712) the RS-11 sum score distribution had a mean of 61.8, a median of 63, and a skewness of -.91. No median differences in resilience scores between males and females (p = .520) were found. Median resilience scores differed between the younger (<72 years) and older (≥72 years) participants (p < .001).

The Kruskal-Wallis test for all participants showed significant differences among age groups (p < .001). This indicates a trend that persons in older age groups were less resilient. This result was confirmed in a stratified analysis of the female (p < .001) and the male groups (p = .008).

### RS-11: reliability and validity

The factor analysis revealed a 1-factor solution prior to rotation via the Kaiser criterion, and scree plot analysis (data not shown). The factor loadings for each item ranged from r = .46 to r = .72. Inter-correlations between items ranged from r = .24 to r = .61 (p < .001). The internal consistency of the RS-11 scale was good (α = .86). AVE for the RS-11 was 19.6% and CR was .76.

Resilience was negatively correlated with depression (r = −.40), anxiety (r = −.32), loneliness (r = −.37), disability (r = −.26), and positively with self-rated health (r = .27) and well-being (r = .36). All correlations were significant (p < .001).

Results of the CFA are given in Table 1. None of the fit indices indicated a good model fit. Thus the unidimensional model of the RS-11 does not fit the data analyzed in this study.

**Table 1:** **CFA of the RS-11 - factor loadings and fit indices (N = 1,856)**

	Factor loadings	<chi > 2 (df)	CMIN/DF	CFI	RMSEA	TLI
**RS-11**	.47-.69	709.219 (44)	16.119	.899	.090	.874

### Development of a shorter scale: RS-5

The “alphamax” macro determined that a minimum of five questions are required to build a scale with a Cronbach’s α > .80. Table 2 lists the 5 possible 5-item scales, which fulfill the internal consistency criteria. Questions G and C are similarly worded, yet they revealed an inter-correlation of r = .60, which is too weak to warrant the exclusion of either question.

**Table 2:** **CFA of abbreviated models – factor loadings and fit indices of 5-item-scales with an internal consistency ≥ 80**

Potential RS-5 models	Factor loadings	<chi > 2 (df)	CMIN/DF	CFI	GFI	RMSEA	TLI	AIC
Model 1: A. B. C. F. G	.67-.69	298.425 (5)	59.685	.899	.936	.178	.799	318.425
**Model 2: C. F. G. H. I**	**.59-.80**	**41.092 (5)**	**8.218**	**.986**	**.991**	**.062**	**.973**	**61.092**
Model 3: B. C. F. G. I	.57-.76	87.153 (5)	17.431	.969	.982	.094	.938	107.153
Model 4: A. B. F. G. K	.60-.73	111.771 (5)	22.354	.960	.976	.107	.919	131.771
Model 5: A. C. F. G. I	.58-.77	125.995 (5)	25.199	.955	.972	.114	.910	145.995

*Abbreviations*: A to I = item number, see Table 1; df = Degrees of freedom; CMIN/DF = Minimum discrepancy, divided by its degrees of freedom; CFI = Comparative-fit index; GFI = Goodness-of-fit-index; RMSEA = Root mean square error of approximation; TLI = Tucker-Lewis-index; AIC = Akaike information criterion. Bold font indicates the model with the best fit indices.

The EFAs of the 5 models revealed a 1-factor solution in each case. This single factor explained 56-58% of the variance.

The uni-dimensional factor solution found in the EFA was subsequently tested for all five shorter versions using CFA, based on the second sub-sample with N = 1,856 participants. Table 2 shows the fit indices and factor loadings of the different 5-item-scales. Regarding all fit indices, model 2 shows the best values in comparison to all other models. Furthermore, the model with the lowest AIC should be preferred, as was the case for model 2. Table 3 summarises all Resilience Scale models.

**Table 3:** **Summary of constructs**

Construct	Mean	Standard deviation	Cronbach’s Alpha	Correlation to RS-11
RS-11	61.8	10.14	.86	
RS-5 Model 1	29.27	5.02	.81	.91
RS-5 Model 2	28.88	4.96	.80	.91
RS-5 Model 3	28.94	4.9	.80	.92
RS-5 Model 4	28.94	5.09	.80	.92
RS-5 Model 5	28.93	5.01	.80	.92

Regarding model 2, all but one fit measure indicated an excellent model fit. The value of CMIN/DF indicates a bad fit, which means a relevant deviation between the data and the model.

Model 2 was tested for invariance across gender and age. As shown in Table 4, the multi-group analyses revealed the invariance of the models across sex and age, because the differences of CFI and RMSEA between the hierarchical nested models are smaller than .01. The < chi > 2 test was nonsignificant for the test of metric invariance, but significant for the test of scalar invariance.

**Table 4:** **Test for invariance across gender and age for model 2**

	N	<chi > 2 (df)	Δ < chi > 2	Δ p	CMIN/DF	CFI	Δ CFI	RMSEA	Δ RMSEA
**Gender**									
Men	863	26.39 (5)			5.28	.98		.07	
Women	993	17.77 (5)			3.55	.99		.05	
**Multigroup analysis**									
Configural model		44.15 (10)			4.42	.99		.04	
Metric model		53.51 (14)	9.36	.053	3.82	.99	.002	.04	.004
Scalar model		81.63 (19)	28.12	<.001	4.3	.98	.008	.04	.003
**Age**									
64-69 years	670	17.87 (5)			3.58	.99		.06	
70-74 years	526	1.66 (5)			0.33	1		.00	
75-79 years	362	24.93 (5)			4.99	.97		.11	
>80 years	298	20.08 (5)			4.02	.96		.10	
**Multigroup analysis**									
Configural model		154.97 (40)			3.874	.956		.039	
Metric model		159.365 (44)	4.39	.36	3.622	.956	.000	.038	.001
Scalar model		190.230 (59)	30.865	.009	3.224	.950	.005	.035	.003

*Abbreviations*: df = Degrees of freedom; CMIN/DF = Minimum discrepancy, divided by its degrees of freedom; CFI = Comparative-Fit index; RMSEA = Root mean square error of approximation.

### RS-5: reliability and validity

Model 2 (including questions C, F, G, H, I) was found to be the best of the tested 5 item versions and further analyses were conducted with this shortened instrument in the full dataset.

For each scale (RS-5 and RS-11) the top tertile of the distribution was classified as resilient to test sensitivity and specificity. There were 221 false positives and 285 false negatives. Falsely classified participants were more likely anxious (OR = 1.5, 95% CI = 1.01 – 2.23) and not lonely (OR = 1.89, 95% CI = 1.24 – 2.87). Furthermore, it distinguished satisfactorily between participants classified as resilient and non-resilient by the RS-11 in all subgroups (Youden’s index ranged from .61 to .77) except in the depressed subgroup.

The RS-5 revealed an internal consistency of α = .80. The single factor explained 57% of the total variance. Factor loadings for items of the RS-5 scale ranged from .61 – .71, inter-item correlations ranged from .39 – .61 (all p < .001). AVE was 25.9% and CR was .70.

The RS-5 scale correlated negatively with the GDS-15 scale (r = −.34), the GAD-7 scale (r = −.29), the loneliness scale (r = −.33), the HAQ-DI (r = −.17), and positively with self rated health (r = .21), all p < .001. The RS-5 correlated strongly with the RS-11 (r = .89, p < .001).

## Discussion

This study has several key findings. Firstly, the validity and reliability of the 11-item Resilience scale (RS-11) as an instrument to measure resilience was confirmed in a large, elderly population. Secondly, a shorter scale with good reliability and validity was developed.

### Reliability and validity of the RS-11 scale

The RS-11 scale was shown to have good internal consistency (α = .86) which is slightly lower than in Schumacher’s analysis (α = .91) (Schumacher et al. 2005). This study reflects previous studies, which showed RS-11 to be a valid instrument compared with the original RS-25 (Schumacher et al. 2005; Rohrig et al. 2006).

Factor loadings for all items of the RS-11 scale were above .50 (except for item d), which indicates that these items loaded significantly to a single factor (Hair et al. 1998).

Correlations with related variables were weak (r = 0.26 - 0.4), but all in the same range as was found in other studies (Wagnild 2009); (Ahern et al. 2006; Schumacher et al. 2005; Wagnild & Collins 2009).

Thus, the unidimensional RS-11, whose validity and reliability as a scale to measure resilience in a population-based sample with a wide age range (14 to 95) has already been shown, has now been confirmed in a population-based sample of elderly individuals (aged 64 to 94).

### Reliability and validity of the RS-5 scale

A shorter scale was developed to encourage its inclusion in large cohort studies and studies in older populations. With regard to the RS-11, being over 72 years old was associated with missing data (OR = 4.54, 95% CI: 2.55 – 8.09). A shorter scale should be less cognitively challenging and easier to administer.

The abbreviated RS-5 satisfies the *a priori* conditions set. CFA of the new RS-5 has confirmed the uni-dimensionality of the questionnaire. All but one fit index indicated that the model fits the data very well. This measure (<chi > 2) is, however, sensitive to sample size. Even a small misspecification would lead to the rejection of the model, and thus we focused on the other fit indices. The RS-5 showed good Youden’s indices for all analyzed subgroups except the depressed subgroup, which had a very small number of cases (n = 65). The RS-5 also correlated strongly with the RS-11 (r = .89, p < .001). The evidence gathered in the development of the RS-11 (Schumacher et al. 2005) put the original 2-factor structure under question. Indeed, a recent review has questioned the methodology behind these dimensions (Windle et al. 2011). Nonetheless, content validity based on the original RS-25 has been preserved, as at least one item from the two originally postulated factor dimensions are represented in the abbreviated scale. Additionally, the measurement invariance of the RS-5 was shown for gender and age, which allows direct comparisons of means between males and females as well as between different age groups.

In conclusion, the RS-5 reproduces the good psychometric properties of the RS-11 in a large, elderly, population-based sample of the German population. Thus, the RS-5 offers a short and easily administered questionnaire, which may be advantageous in large cohort studies with extensive tests for participants and in studies with older individuals.

### Study limitations

Due to the cross-sectional design of this study it was not possible to calculate test-retest reliability of either the RS-5 or the RS-11. No concurrent validity was measurable in this study since no other instrument measuring resilience was included. An optimal assessment of resilience would be conducted via clinical interview; however, for the purposes of large cohort studies, the telephone interview is the preferred method.

### Future aims

Further investigations regarding the test-retest reliability and the potential for use in other ethnic groups of both the RS-11 and RS-5 scales are warranted. Validation of the RS-5 in younger age groups is also desirable. The shortened RS-5 scale was incorporated into the next stage of the KORA – Age cohort study, allowing for a wide range of longitudinal analyses to determine predictors for and health outcomes of resilience.

## Conclusions

In conclusion, the RS-11 and RS-5 were shown to be valid and reliable instruments to measure resilience in an elderly German population. The RS-5 displays excellent model fit statistics and distinguishes well between participants classed as resilient by the RS-11 and is thus a highly recommended scale for measuring resilience.

## Electronic supplementary material

Additional file 1: **Resilience was measured using the German RS-11 scale.** (DOC 26 KB)

## Abbreviations

RS-11: Resilience scale – 11 items
CFA: Confirmatory factor analysis
KORA: Kooperative Gesundheitsforschung in der region Augsburg
MONICA: MOnitoring trends and determinants in cardiovascular disease
TICS-m: Telephone interview for cognitive status - modified
OR: Odds ratio
CI: Confidence interval
RS-25: Resilience Scale – 25 items
GDS-15: Geriatric depression scale −15 items
GAD-7: Generalized anxiety disorder scale
WHO: World health organization
SNI: Social network Index
MOPSY: WHO: MONICA psychosocial optional study
HAQ-DI: Health assessment questionnaire disability index
EFA: Exploratory factor analysis
AVE: Average variance extracted
CR: Composite reliability
CMIN/DF: Minimum discrepancy, divided by its degrees of freedom
CFI: Comparative-fit index
GFI: Goodness-of-fit-index
RMSEA: Root mean square error of approximation
TLI: Tucker-Lewis-index
AIC: Akaike information criterion.
