# Supplementary material for: Validation and development of a shorter version of the resilience scale RS-11: results from the population-based KORA–age study
Source: BMC Psychol. 2013 Nov 22;1(1):25. doi: 10.1186/2050-7283-1-25 (PMC4270035; doi:10.1186/2050-7283-1-25)
Supplement: Supplementary file 1 — Additional file 1: Resilience was measured using the German RS-11 scale. (DOC 26 KB) [file 40359_2013_20_MOESM1_ESM.doc]

**Additional File 1.** RS-11

Ich lese Ihnen nun ein paar Feststellungen vor. Bitte sagen Sie mir, wie sehr die Aussagen im Allgemeinen auf Sie zutreffen, d.h. wie sehr Ihr übliches Denken und Handeln durch diese Aussagen beschrieben wird.

*Wir haben eine Skala von 1 bis 7. 1 bedeutet „Nein, ich stimme nicht zu“. 7 bedeutet „Ja, ich stimme völlig zu.“ Sie können ebenso einen Wert dazwischen wählen.*

I shall read a few statements to you. Please tell me how much you agree with the statements in general, i.e. how well your usual thinking and actions are described by these statements.

We have a scale from 1 to 7. 1 means “no, I disagree.” 7 means "I agree completely.” You may choose any value from 1 to 7.

- - 1. Wenn ich Pläne habe, verfolge ich sie auch.1 2 3 4 5 6 7

When I make plans I follow through with them.

- - 1. Normalerweise schaffe ich es irgendwie. 1 2 3 4 5 6 7

I usually manage one way or another.

- - 1. Es ist mir wichtig, an vielen Dingen interessiert zu bleiben. 1 2 3 4 5 6 7

Keeping interested in things is important to me.

- - 1. Ich mag mich. 1 2 3 4 5 6 7

I am friends with myself.

- - 1. Ich kann mehrere Dinge gleichzeitig bewältigen. 1 2 3 4 5 6 7

I feel that I can handle many things at a time.

- - 1. Ich bin entschlossen. 1 2 3 4 5 6 7

I am determined.

- - 1. Ich behalte an vielen Dingen Interesse. 1 2 3 4 5 6 7

I keep interested in things.

- - 1. Ich finde öfter etwas, worüber ich lachen kann. 1 2 3 4 5 6 7

I can usually find something to laugh about.

- - 1. Normalerweise kann ich eine Situation aus mehreren Perspektiven betrachten.

1 2 3 4 5 6 7

I can usually look at a situation in a number of ways.

- - 1. Ich kann mich auch überwinden, Dinge zu tun, die ich eigentlich nicht machen

will. 1 2 3 4 5 6 7

Sometimes I make myself do things whether I want to or not.

- - 1. In mir steckt genügend Energie, um alles zu machen, was ich machen muss.

1 2 3 4 5 6 7

I have enough energy to do what I want to do.
